# Peaceful Death in Japanese YouTube Videos: Content and Network Analysis

**DOI:** 10.2196/81861

**Published:** 2026-03-13

**Authors:** Xanat Vargas Meza, Masanori Oikawa

**Affiliations:** 1 Institute for the Advanced Study of Human Biology, Kyoto University, Yoshida-Konoe-cho, Sakyo-ku, Kyoto, 606-8501, Japan, 81 08091707764; 2 Department of Medical Ethics, Tohoku University Graduate School of Medicine, Sendai, Japan

**Keywords:** end-of-life, family networks, Japan, social media, YouTube

## Abstract

**Background:**

Death is a difficult topic to discuss for many. Notwithstanding, there is much to learn regarding the contemporary Japanese people’s views on a good (peaceful) death. Particularly, shifts in public perceptions of death following the beginning of the COVID-19 pandemic should be considered by health care staff who deliver end-of-life care.

**Objective:**

This study examined the recent representations of peaceful death in Japanese YouTube videos to understand related narratives and family structures.

**Methods:**

We examined 457 YouTube videos about peaceful death using content and family network analyses. The videos were classified into 3 groups: ordinary people, celebrities, and fictional characters.

**Results:**

We identified emerging medical actors who discussed end-of-life with the public. Death portrayed or discussed in the videos mostly involved adults aged 18 to 59 years (179/501, 36% ages), and the most common narrative was illness (101/839, 12% narratives). Although the most common religious stance was nonreligious (304/532, 57.14% religious stances), videos of regular people tended to mention or show a religious practice or symbol (154/272, 56.61% religious stances). There were significant differences (*F*_2, 454_=22.81, *P*<.001) in terms of gender between celebrity and regular videos (*P*<.001, 95% CI 0.23-0.49), celebrity and fiction videos (*P*=.005, 95% CI 0.05-0.34), and fiction and regular videos (*P*=.008, 95% CI 0.03-0.29). This suggests a bias towards male representations of death among celebrities and fictional characters. Male family members were also overrepresented in videos about celebrities and fictional characters as per the visualization of family networks. The networks also suggested that common people’s families were more complex than celebrity and fictional people’s families. Furthermore, in common people’s families, women and men were equally important when reaching out to other family members quickly and in terms of trust.

**Conclusions:**

The current image of Japan as a nonreligious nation, and the gender bias in Japanese social media should be challenged. Furthermore, health care and research protocols related to end-of-life care for patients beyond older adults should be developed.

## Introduction

### Background

Many find death to be a difficult topic to discuss in contemporary times, and our treatment of death impacts how we approach disability and sickness. Death may have several causes, and people adopt various attitudes according to previously agreed-upon values surrounding death. In general, Japanese people avoid talking about death. In Japan, since most people die in hospitals [1], a “good death” in medical contexts involves a grasp on diagnoses, treatment options, and whether patients and relatives decide the place to be treated or laid to rest [2]. This concept has been developed through interactions between medical staff, patients, and their kin and is linked to cultural and spiritual values [3,4]. However, not much is known about “good death” in other contemporary Japanese contexts.

In a previous study, we examined the concept of good death as a “peaceful death” on Japanese Twitter (subsequently rebranded X; Meza Xanat and Masanori, unpublished data). We found it a useful means to uncover conversations about difficult topics, as social media functions with a layer of anonymity that allows more free, open, and spontaneous discussions than conventional surveys and interviews [5]. As Japanese Twitter users talking about death tend to be young, we turned our attention to YouTube to complement previous findings. Launched in 2005, this video social media platform is the second most visited website after Google, with broader advertising revenue for male content creators (51.8% vs 48.2%) and broader advertising reach for male viewers (76.4% vs 66.1%, overlapped data) [6]. Additionally, it appears to appeal to a wide age range of Japanese users [7].

Therefore, our objective in this study was to examine public communication and perceptions of “good death” on Japanese YouTube. We reflected on what is considered a good death and end-of-life preparations.

### Narratives of Death in Japan

Less educated people, Soka Gakkai Buddhists, and women have a greater tendency to practice religion and believe in the afterlife than others [8]. In Yamaguchi in Honshu (the largest island of Japan), 49% of the rural people interviewed had no image associated with the afterlife, recognized a continuity between life and death, and confronted it in a collective manner [9]. Meanwhile, a study found that the public does not know about the afterlife, and medical staff mostly do not believe in it; the colors associated with death were gray, white, and black; and this signaled a need for education on death [10].

Long [3,11] conducted interviews on the Japanese perception of death in hospitals and hospices, including religious themes. Patients’ experience of dying, autonomy, social context, control over the dying process, and quality of end-of-life care were relevant for a good death [4]. A study on patients with cancer reported their desire to fight the disease, retain hope, unawareness of death, and prioritizing good relationships with family; whereas those who did not want to fight cancer prioritized physical and mental comfort during end-of-life care [12]. Morooka [13] found that the families of those who died at home focused on daily life and dying without causing care or financial burdens. Finally, Gould [14] noted that end-of-life products and services by Buddhism-related actors in Japan cater to lonely, stressed families through healing (*iyashi*) and a calm heart.

Regarding social media, Sueki [15] correlated clear and specific statements of wanting to commit suicide on Twitter with suicidal behavior, but not general conversations or mentions of death. Social learning theory argues that media consumption reinforces notions of what is acceptable, including suicide [16]. This theory has also been applied to celebrity studies. Katsumata [17] proposes that deaths by accident, epidemics, or environmental disasters on social media should convey the gravity of lives lost and the associated grief, with previous consent of the bereaved kin.

### Death in Video-Based Social Media Platforms

Technological tools such as photography and films are not neutral. As much as they capture and recreate beauty, they can also be used to export ideas of colonialism, empire, and White supremacy, objectifying communities and societies in the context of death [18]. Varied intentions in capturing or manipulating records are not new features of video shared on social media. Furthermore, the scale and potential reach of such materials have increased due to the high rate of internet service adoptions.

Some of the earliest examinations of death on YouTube were tied to crime [19,20], which highlights the sensationalization of tragedies. Celebrity deaths were also reported [21,22]. Furthermore, 377 YouTube videos showcased framing by organ procurement organizations [23]. The music in videos featuring mourning on YouTube elicited empathy and sadness [24], even when in the background [25].

Ethnography of a multilingual YouTube video for children regarding death and grief noted that the decade-old video went viral across Spanish-speaking countries at the beginning of the COVID-19 pandemic, reaching all genders and ages, and this highlighted the gravity of sudden death in a manner that official information sources did not [26]. Content analysis of 100 videos on grief on TikTok showed that users grieved openly and emotionally [27]. Interviews with grievers during the COVID-19 pandemic, including 3 YouTube users, showed that they announced the loss, shared artistic and farewell posts, and felt that this helped them maintain a link with the deceased; however, in cases of prolonged grieving, other social media users perceived them to be insincere [28].

Regarding Japan, an analysis of 27 English YouTube videos about the Minamata disease noted that most uploaders were ordinary people using documentary clips, lacking context and explanations [29]. Uriu et al [30] published a case study of a funeral live cast on YouTube, making it more accessible to older people kin who lived far away; however, they argued that the process could be enriched further through other data formats, and there was a need to consider religious differences. A comparison of the mourning of Asian virtual YouTube uploaders with human and celebrity uploaders revealed that young viewers treated humans more like machines [31]. Finally, a study of suicidal ideation in 28 Twitter and YouTube vlogs by women revealed empathic messages, and that the belief that the ability to mitigate suicidal ideation lies in the individual has not changed in the last decade [32].

We found that studies analyze anything from 1 to over a 1000 videos, there is little research on Japanese video-based social media and death, and studies on religious narratives outside of Buddhism are lacking. Therefore, we formulated the following research questions (RQs) in the context of Japanese YouTube videos:

1. RQ1: Who are involved in creating and appearing in death-related content?
2. RQ2: What are the most common narratives regarding death?
3. RQ3: Which religions are associated with death?

### Family Networks

A social network is a set of nodes representing actors (eg, persons) and their ties (relationships). Similarly, a family network is a representation of family members and their relationships. Given that the study of death in social media has not focused on end-of-life care, and that kin are crucial in making decisions in this stage in Japan, we reviewed the literature on family networks.

Social network methods broaden the definition of family by starting with individuals’ definition of their family context, and key applications include national representative survey data, family networks across time, and family health networks [33]. Recent research on family networks at the end of life has been conducted primarily in Australia. A survey among 7915 individuals over a period of 7 years indicated that care providers were of all adult ages, more than half were extended family and friends, and dying at home was common in these contexts [34]. Examining care networks across time in 9 focus groups of people dying at home revealed that these networks grow and relationships strengthen [35]. A similar study integrating surveys revealed that relationships with medical care staff were peripheral and those between staff were weak [36]. Finally, network data from an American survey conducted over a 5-year period clarified that older adults tended to substitute dead confidants more frequently than younger adults [37].

In Japan, a survey of 129 families in Tokyo identified 11 family structures and the important role of matrilineal and nonkin ties [38]. Maeda and Meguro [39] examined the strength of urban family ties, noting the influence of class. A comparison between the family networks of older men in urban and rural settings over a 7-year period revealed that mortality increased when rural men were less educated or unmarried; and this also applied to urban men who did not participate in community groups [40]. Ishiguro [41] noted that the size of kin and friendship networks slightly decreased among women, and for men, networks comprising reliable friends reduced slightly between the 1990s and 2010s. In addition, a survey among 355 older patients in Fukushima showed that those with small social networks discussed end-of-life plans less, regardless of marital status [42].

Although we identified literature on networks in Japan, family networks during the COVID-19 pandemic remained unexplored. Therefore, we added the following RQs for the portrayal of death in Japanese YouTube videos:

1. RQ4: Which kin were involved in death-related content?
2. RQ5: What structures do family networks display during the end-of-life period?

## Methods

### Data Collection and Organization

We tested several keywords based on narratives about “good death” in Japan [3] in Google trends and YouTube. We selected ‘安らかな死’ (peaceful death) as a proxy for good death in medical and sociocultural contexts because of the high number of hits. A researcher extracted video data using YouTube Data Tools [43], in 2 modules that use the YouTube Application Programming Interface. The Video List module retrieves a list of videos and related information (identifier, publication date, title, description, tags, category, duration, number of views, and number of comments). The video titles were reviewed and the videos were watched to identify those related to death. This resulted in 457 out of 2074 videos, published from January 2019 to May 2023. Subsequently, a researcher used identifiers (the last part of the video hyperlink) obtained through the Video List module to extract comments using the Video Comments module.

### Content Analysis

The content analysis was divided into 3 parts: human notation of video content, automatic summarization of video descriptions, and automatic summarization of video comments.

#### Human Notation of Video Content

The 457 videos were watched and annotated, based on previous studies [44,45] as follows:

- To answer RQ1, a researcher identified the gender of the deceased and sorted them according to age: child (0-17 y), adult (18-59 y), and older adult (≥60 y). The “About” page of the YouTube channel was consulted to identify uploaders, and speakers were considered individuals who appeared, spoke, or whose words were conveyed in the video. These were then categorized (Multimedia Appendix 1).
- To answer RQ2, a researcher used the location information on the channel’s “About” page and from within the YouTube video. This information was categorized according to countries and Japanese regions. Furthermore, death and end-of-life narratives were classified based on prior studies (Multimedia Appendix 2) [7,9,10,45-47].
- To answer RQ3, we considered religion as supernatural beliefs about death and the afterlife, and activities surrounding death. Therefore, the religions mentioned, religious buildings, religious imagery, and rites displayed in the videos were annotated.
- To answer RQ4 and RQ5, kin was annotated. A sample of videos with kin information (73/164, 44.51%) was verified by a second researcher. Their agreement on family members ranged between 0.83 and 1 (Multimedia Appendix 3), and the corrected notations were incorporated into the data.

#### Automatic Summarization of Video Descriptions

To answer RQ1 and RQ2, we used KH Coder (Ritsumeikan University) [48] to summarize frequently appearing terms in Japanese and their relationships with other terms, generating co-occurrence–based networks. The networks were drawn as undirected and unipartite, and overlap coefficients were calculated in KH Coder. In the overlap coefficient between 2 groups of words, when group X was a subgroup of Y, the overlap coefficient was equal to 1 [49]. We used this coefficient for its capability to generate unified networks wherein word groups are still distinct. As recommended by Higuchi [50], the 60 strongest co-occurrences were drawn as network edges.

#### Automatic Summarization of Video Comments

To complement the response to RQ2, Kh Coder was used to summarize the most frequent words in the video comments.

### Statistical Analysis

To address RQ1-RQ4, the videos were divided into 3 groups: death of a (real) regular person, death of a celebrity, and fictional death. Each parametric numerical variable that had a median over zero (speakers, religion, and deceased disclosed gender) was analyzed through a one-way ANOVA. We considered a threshold of *P*<.01 as significant. All statistical tests were run in IBM SPSS (version 29.0.10).

### Network Analysis

Network analysis involves quantitative and qualitative tools. To answer RQ 5, we focused on measurements at the node and network level based on prior studies [51-54]. While many social network studies focus on trust, Yamagishi [55] argues that Japanese people seek assurance when creating stable relationships. Thus, we considered trust as equivalent to assurance in our study. The network measurements (Multimedia Appendix 4) were calculated using Gephi version 0.10.1 [56,57]. Gephi was also used to draw unipartite undirected networks laid with the Fruchterman-Reingold algorithm of family member relationships found among the regular people, celebrities, and fictional characters.

### Ethical Considerations

This study was exempt from ethical review by the Medical Board of Kyoto University because it involved big data analysis of social media records and did not use human data beyond measuring internet activity. This study follows the Ethical Guidelines for Internet Research [58] and the Ethical Guidelines for Medical and Biological Research Involving Human Subjects [59]. Based on the above, it was determined that the study did not require informed consent from participants. The findings pertaining to nonpublic persons, including those related to medical conditions, were reported in a way that masks identities.

## Results

### Actors in Peaceful Death

Table 1 displays the overlapping 477 uploaders found on the 457 YouTube videos, divided in videos of regular people, celebrities, and fictional characters. No significant differences were found between video groups. Among the 477 uploaders, 208 (43.60%) were citizens, 65 (13.62%) the media, and 44 (9.22%) religious actors.

**Table 1.** YouTube uploaders in 457 videos.

Actor	Regular videos (n=220), n (%)	Celebrity videos (n=122), n (%)	Fictional videos (n=115), n (%)	Total videos (N=457), n (%)
Doctor	22 (10)	0 (0)	0 (0)	22 (5)
Medical student	0 (0)	0 (0)	0 (0)	0 (0)
Nurse	1 (0)	0 (0)	0 (0)	1 (0)
Other medical staff	5 (2)	0 (0)	0 (0)	5 (1)
Donor	0 (0)	0 (0)	0 (0)	0 (0)
Recipient	0 (0)	0 (0)	0 (0)	0 (0)
Donor relative	0 (0)	0 (0)	0 (0)	0 (0)
Recipient relative	0 (0)	0 (0)	0 (0)	0 (0)
Other deceased	1 (0)	1 (1)	1 (1)	3 (1)
Other deceased relative	4 (2)	0 (0)	0 (0)	4 (1)
Association	9 (4)	1 (1)	1 (1)	11 (2)
Citizen	91 (41)	50 (41)	67 (58)	208 (46)
Government	2 (1)	0 (0)	0 (0)	2 (0)
Media	26 (12)	31 (25)	8 (7)	65 (14)
Religious actor	36 (16)	4 (3)	4 (3)	44 (10)
Educational actor	5 (2)	0 (0)	0 (0)	5 (1)
Unknown	34 (15)	36 (30)	37 (32)	107 (23)
Total overlapping actors	236 (107)	123 (101)	118 (103)	477 (104)

Regarding the 1303 overlapping speakers (Table 2), 355 (27.24%) were nonorgan donor deceased, 329 (25.24%) were citizens, and 187 (14.35%) were the kin of nonorgan donor deceased. There were significant differences (*F*_2, 454_=5.72; *P*=.001) in terms of nondonor deceased between the videos on regular people and celebrities (*P*=.02, 95% CI 0.02-0.24), and those of regular people and fictional characters (*P*=.02, 95% CI 0.02-0.25). There was also a difference (*F*_2, 454_=8.75; *P*<.001) between videos of regular people and fictional characters in terms of citizens (*P<*.001, 95% CI 0.09-0.33). This implies that nondonor deceased and citizens are less present in regular people death’s videos.

**Table 2.** Speakers in 457 YouTube videos.

Actor	Regular videos (n=220), n (%)mean, standard deviation	Celebrity videos (n=122), n (%) mean, standard deviation	Fictional videos (n=115), n (%) mean, standard deviation	Total videos (N=457), n (%) mean, standard deviation
Doctor	62 (28) 0,28, 0.45	8 (6) 0.06, 0.24	13 (11) 8.85, 0.31	83 (18) 0.18, 0.38
Medical student	0 (0) 0.00, 0.00	0 (0) 0.00, 0.00	0 (0) 0.00, 0.00	0 (0) 0.00, 0.00
Nurse	20 (9) 0.09, 0.28	3 (2) 0.02, 0.15	8 (7) 0.07, 0.25	31 (7) 0.07, 0.25
Other medical staff	15 (7) 0.07, 0.25	1 (1) 0.01, 0.09	1 (1) 0.01, 0.09	17 (4) 0.04, 0.18
Donor	0 (0) 0.00, 0.00	0 (0) 0.00, 0.00	1 (1) 0.01, 0.09	1 (0) 0.00, 0.04
Recipient	0 (0) 0.00. 0.00	0 (0) 0.00, 0.00	1 (1) 0.01, 0.09	1 (0) 0.00, 0.04
Donor relative	0 (0) 0.00. 0.00	0 (0) 0.00, 0.00	1 (1) 0.01, 0.09	1 (0) 0.00, 0.04
Recipient relative	0 (0) 0.00. 0.00	0 (0) 0.00, 0.00	1 (1) 0.01, 0.09	1 (0) 0.00, 0.04
Other deceased	156 (71) 0.71, 0.45^a^	102 (84) 0.84, 0.37	97 (84) 0.84, 0.36^a^	355 (78) 0.78, 0.41
Other deceased relative	114 (52) 0.52, 0.50	24 (20) 0.20, 0.39	49 (43) 0.43, 0.49	187 (41) 0.41, 0.49
Association	13 (6) 0.06, 0.23	1 (1), 0.01, 0.09	1 (1) 0.01, 0.09	15 (3) 0.03, 0.17
Citizen	141 (64)^a^ 0.64, 0.48	90 (74) 0.74, 0.44	98 (85) 0.85, 0.35	329 (72) 0.72, 0.45
Government	47 (21) 0.21, 0.41	16 (13) 0.13, 0.33	41 (36) 0.36, 0.48	104 (23) 0.23, 0.42
Media	27 (12) 0.12, 0.32	37 (30) 0.30, 0.46	3 (3) 0.03, 0.16	67 (15) 0.15, 0.35
Religious actor	50 (23) 0.23, 0.42	9 (7) 0.07, 0.26	11 (10) 0.10, 0.29	70 (15) 0.15, 0.36
Educational actor	26 (12) 0.12, 0.26	6 (5) 0.05, 0.21	7 (6) 0.10, 0.24	39 (9) 0.10, 0.28
Unknown	0 (0) 0.00, 0.29	1 (1) 0.01, 0.24	1 (1) 0.01, 0.09	2 (0) 0.00, 0.06
Total overlapping actors	671 (305) 3.05	298 (244) 2.44	334 (290) 3.00	1303 (285) 2.85

a Statistically significant results.

The most common age group in 501 overlapped ages in the 457 videos was adults (n=179, 36%), followed by the unknown group (n=158, 31%), older adults (n=121, 24%), and children (n=43, 9%). For regular people, age went mostly unreported (99/256, 39%), whereas for celebrities and fictional characters, most deaths were of adults (52/122, 42% and 59/123, 48% respectively). Regarding gender, Table 3 shows that in 262 of the 550 (47.63%) overlapped genders identified, the deceased were male. There were significant differences (*F*_2, 454_=22.81; *P<*.001) in terms of gender between celebrity and regular videos (*P<*.001, 95% CI 0.23-0.49), celebrity and fiction videos (*P*=.005, 95% CI 0.05-0.34), and fiction and regular videos (*P*=.008, 95% CI 0.03-0.29). This supports the notion that most male deaths were those of celebrities, followed by fictional characters.

**Table 3.** Overlapped perceived gender of the deceased.

Perceived gender of the deceased	Regular videos (n=220), n (%) mean, standard deviation	Celebrity videos (n=122), n (%) mean, standard deviation	Fictional videos (n=115), n (%) mean, standard deviation	Total videos (N=457), n (%) mean, standard deviation
Men	96 (44) 0.44, 0.49^a^	97 (80) 0.80, 0.40^a^	69 (60) 0.60, 0.49^a^	262 (57) 0.57, 0.49
Women	87 (40) 0.40, 0.49	30 (25) 0.25, 0.43	53 (46) 0.46, 0.50	170 (37) 0.37, 0.48
Other or unknown	90 (41) 0.41, 0.49	2 (2) 0.02, 0.12	26 (23) 0.23, 0.42	118 (26) 0.26, 0.43
Total perceived gender of the deceased	273 (124) 1.24	129 (105) 1.05	148 (128) 1.28	550 (120) 1.20

a Statistically significant results.

### Narratives of Peaceful Death

This section presents the contexts of peaceful deaths described in the videos to address RQ2. The most common country identified in overlapping data was Japan (384/592, 64.86%), and a significant difference (*F*_2, 454_=6.44; *P*=.002) was found between regular people and celebrity videos (*P*=.001, 95% CI 0.05-0.28). This implies that most of the celebrity deaths highlighted in the Japanese videos occurred abroad.

**Figure 1.**
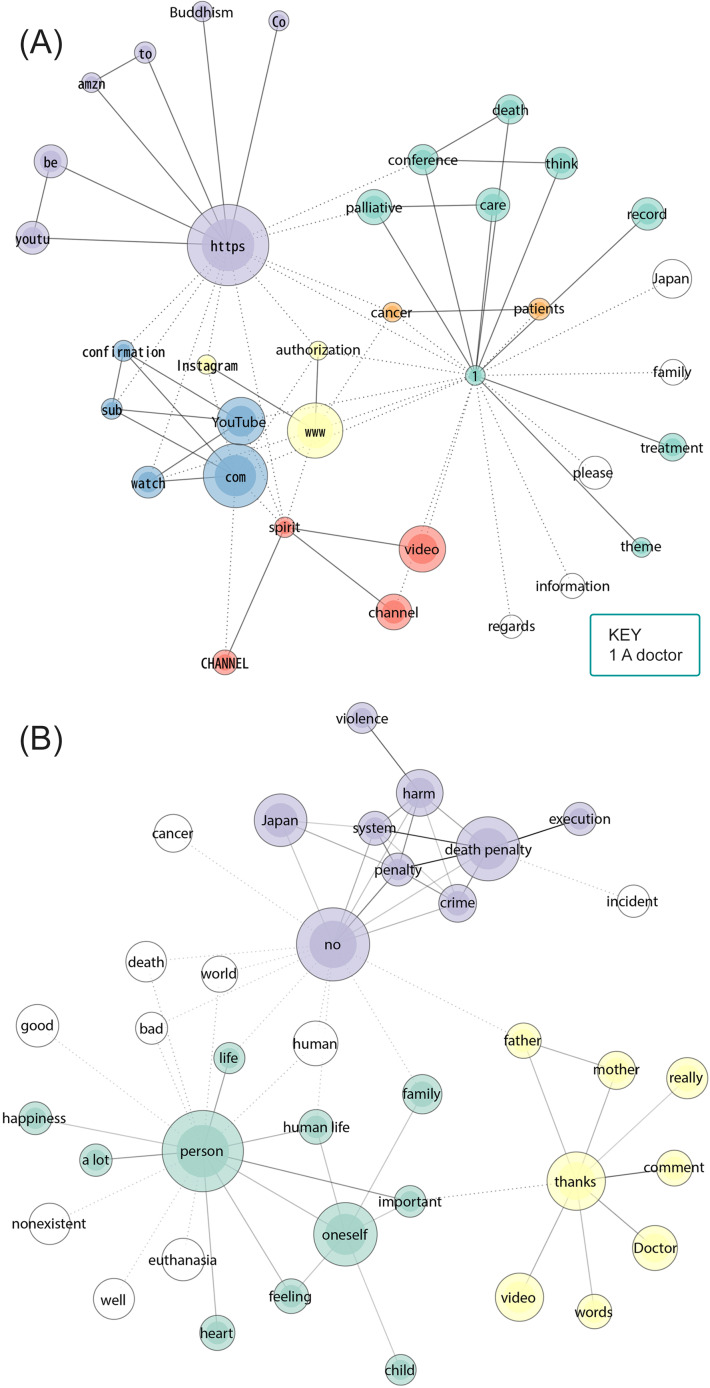
(A) Descriptions (word frequency 50‐1356; tie strength 0.6‐1) and (B) comments (word frequency 900‐6649; tie strength 0.3‐0.6) of YouTube videos about ordinary people’s deaths.

**Figure 2.**
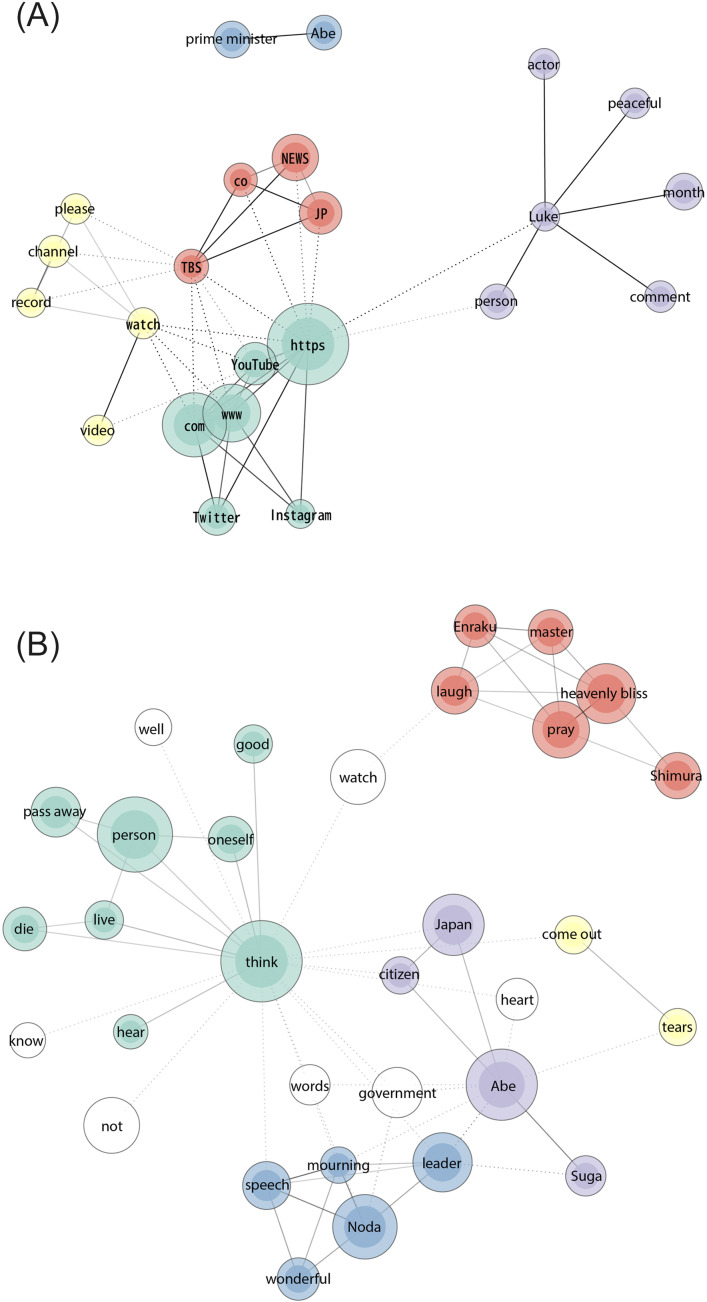
(**A**) Descriptions (word frequency 34‐282; tie strength 0.8‐1) and (**B**) comments (word frequency 600‐3486; tie strength 0.4‐0.8) of YouTube videos about celebrities’ deaths.

**Figure 3.**
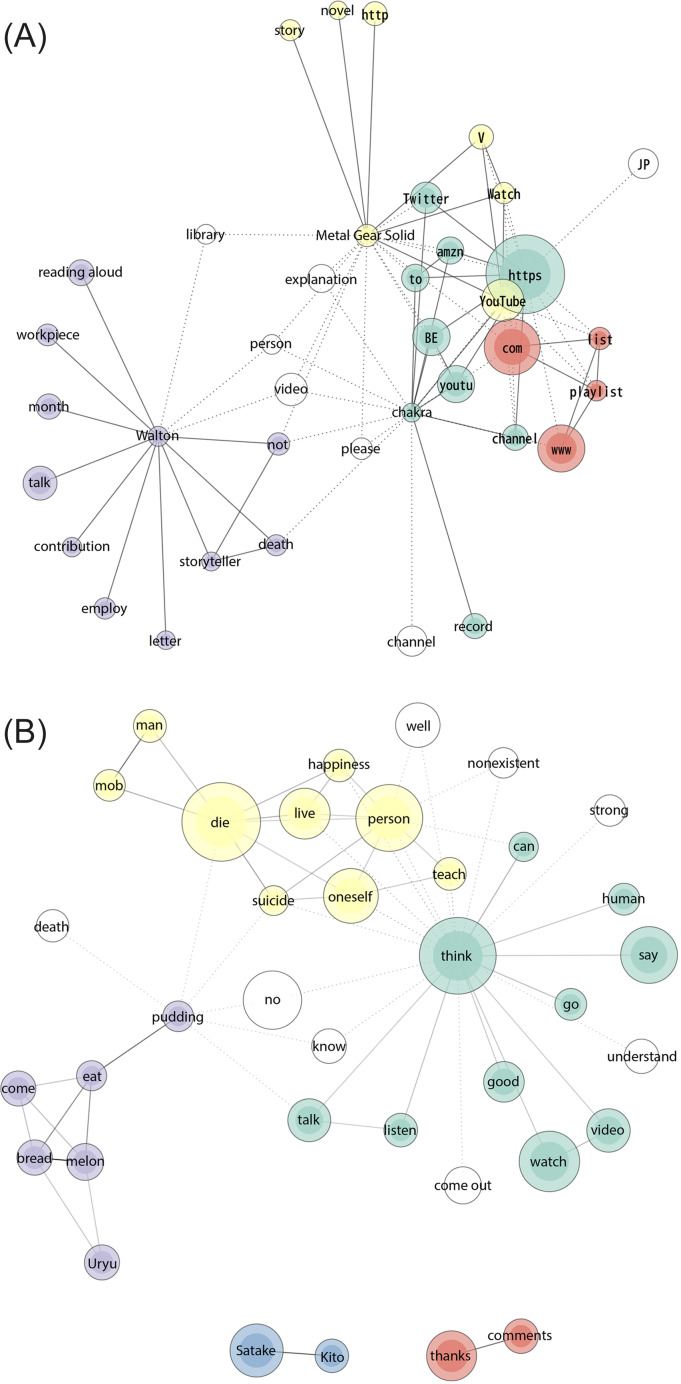
(**A**) Descriptions (word frequency 30‐577, tie strength 0.6‐1) and (**B**) comments (word frequency 300‐2260, tie strength 0.2‐0.8) of YouTube videos about fictional characters’ deaths.

Figures 1-3 display the communication channels of peaceful death. The node size reflects word frequency, with thicker lines indicating stronger tie strengths between words. Nodes with the same color indicate that they belong to the same group, and nodes with no color are not part of a specific group. Figure 1A shows descriptions of regular people’s deaths, where a purple cluster of words wherein Buddhism is connected to the Amazon store and other YouTube videos. The red cluster refers to descriptions found in videos related to spiritualism and Nnew Rreligion (the term “spirit”), linked to YouTube video channels. Figure 2A shows that celebrity deaths were communicated through YouTube, Twitter, Instagram, and the Tokyo Broadcasting System (TBS; in a red cluster). By contrast, Figure 3A shows only YouTube and Twitter.

A summary of the descriptions of regular people’s videos in Figure 1A is shown in green, referring to end-of-life care in hospices and academic conferences. Although less central, there seems to be a focus on patients with cancer (orange cluster). Regarding the comments on these videos, Figure 1B shows a green cluster discussing life, family, and families. The second cluster (in yellow) expresses gratitude to medical experts for their insights, mentioning bereaved parents (“father” and “mother”). Peaceful death seems to be connected to medical death and death in hospices. The purple cluster and some loosely connected words include what is considered the opposite of peaceful death, for instance, the death penalty and euthanasia.

The descriptions of celebrities shown in Figure 2A centered around 2 men: American actor Luke Perry (purple cluster) and Japanese politician Shinzo Abe (blue cluster). There was a contrast between the formal language used for Abe and the emotional language used for Perry (“peaceful”). The video comments shown in Figure 2B were mostly about Abe’s death (purple cluster), the official response of the opposition party (blue cluster), and a former political rival (Suga in the purple cluster). There were prayers for 冥福 (heavenly bliss, identified 1876 times in the red cluster) for the deaths of rakugo (traditional comedy) performers Shimura and Enraku. This is a common praying behavior in Buddhist funerals. Conversely, the word “paradise” related to Christian beliefs was found 510 times (not shown in the figure).

As for fictional deaths, the most central word group of descriptions shown in Figure 3A is based on a video about a Twitter webtoon called “*The Crocodile Who Dies After 100 Days*.” This webtoon was tied to New Age beliefs (the word “chakra”). A cluster in yellow shows the term “Metal Gear Solid,” an adventure stealth video game franchise connected to a “novel.” A group in purple shows a character (“Walton”) from *Frankenstein* by Mary Shelley and other words from videos where people read novels aloud. Regarding the video comments, although fictional deaths had various causes, most focused on suicide (yellow cluster in Figure 3B). The yellow and purple groups were comments on a webtoon that humanized a fictional former mob man. Satake and Kito (blue cluster) are also characters in a webtoon. Overall, conversations are focused on the opposite of a good death, that is, on violent deaths.

Regarding the annotated narratives that complement these figures, about a quarter (n=222, 26%) of the overlapping 839 narratives explained death, and 101 (12%) focused on illness (Multimedia Appendix 5).

### Religion and Peaceful Death

In reference to RQ3, Table 4 suggests that the most identified religion stance in the overlapping data was nonreligious (304/532, 57.14%). There was a significant difference (*F*_2, 454_=23.58; *P<*.001) in religion between celebrity and regular videos (*P*<.001, 95% CI 0.23-0.47). This suggests that religious practices were most prominent in regular people’s deaths. Table 4 notes Buddhism as the most common religion, although a wide variety of religions were found. Other Asian religions included Ainu beliefs, Brahmanism, Confucianism, Feng shui, Hinduism, Onmyodo, Ryukyuan beliefs, Taoism, and beliefs of the Indigenous people in China. Other Christian groups included Evangelists, Protestants, and unspecified Christian beliefs. Finally, other Middle Eastern and Western religions included Greek paganism, Islam, Judaism, and unspecified Abrahamic beliefs.

**Table 4.** Overlapping religions in 457 videos about peaceful death.

Religion	Regular videos (n=220), n (%) mean, standard deviation	Celebrity videos (n=122), n (%) mean, standard deviation	Fictional videos (n=115), n (%) mean, standard deviation	Total videos (N=457), n (%) mean, standard deviation
Animism or spiritualism	7 (3) 0.03, 0.17	0 (0) 0.00, 0.00	3 (3) 0.03, 0.16	10 (2) 0.02, 0.14
Catholic Christianity	12 (5) 0.05, 0.22	0 (0) 0.00, 0.00	1 (1) 0.01, 0.09	13 (3) 0.03, 0.16
Other Christianity	24 (11) 0.11, 0.31	1 (1) 0.01, 0.09	4 (3) 0.03, 0.18	29 (6) 0.06, 0.24
Japanese Buddhism	14 (6) 0.06, 0.24	2 (2) 0.02, 0.12	0 (0) 0.00, 0.00	16 (4) 0.04, 0.18
Other Buddhism	48 (22) 0.22, 0.41	9 (7) 0.07, 0.26	26 (23) 0.23, 0.42	83 (18) 0.18, 0.38
New religions	14 (6) 0.06, 0.24	4 (3) 0.03, 0.17	0 (0) 0.00, sd	18 (4) 0.03, 0.19
Other Asian religions	11 (5) 0.05, 0.21	1 (1) 0.01, 0.09	5 (4) 0.04, sd	17 (4) 0.04, 0.18
Other Middle East or Western religions	9 (4) 0.04, 0.19	0 (0) 0.00, 0.00	2 (2) 0.02, sd	11 (2) 0.02, 0.15
Shinto	15 (7) 0.07, 0.25	1 (1) 0.01, sd	15 (13) 0.13, 0.33	31 (7) 0.07, 0.25
No religion	118 (54) 0.54, 0.50*^a^	108 (89) 0.89, 0.32*	78 (68) 0.68, 0.46	304 (67) 0.67, 0.46
Total of religion stances	272 (123) 1.23	126 (103) 1.03	134 (116) 1.16	532 (116) 1.16

a Asteriscs indicate the results are statistically significant

### Family Networks of Those Deceased

To address RQ6, Table 5 displays the network measurements of 164 videos that mentioned family members. Please refer to Multimedia Appendix 4 to interpret these results. The high number of edges in the regular and fictional families implies trust (assurance). This is reinforced with the highly weighted degree and high clustering coefficient in the networks of regular people. Access to other family members is faster in regular families based on closeness and path length. Regular family structures are the densest and most complex, as supported by density and the number of triangles. These structures are also the most unified based on their low modularity.

**Table 5.** Network measurements of family networks calculated with Gephi.

Measure	All videos	Regular	Celebrity	Fictional
Nodes	17	13	10	14
Edges	62	30	19	32
Average weighted degree	47.529	40.6150	8.8000	12.7140
Average harmonic closeness	0.7279	0.8012	0.7111	0.6758
Average betweenness	0.0362	0.0361	0.0722	0.0540
Average clustering coefficient	0.8430	0.8830	0.7810	0.8090
Average path length	1.5441	1.3970	1.5777	1.6483
Density	0.456	0.6030	0.4220	0.3520
Diameter	2	2	2	2
Modularity	0.037	0.022	0.047	0.127
Triangles	118	96	14	32
Connected components	1	1	1	1

**Figure 4.**
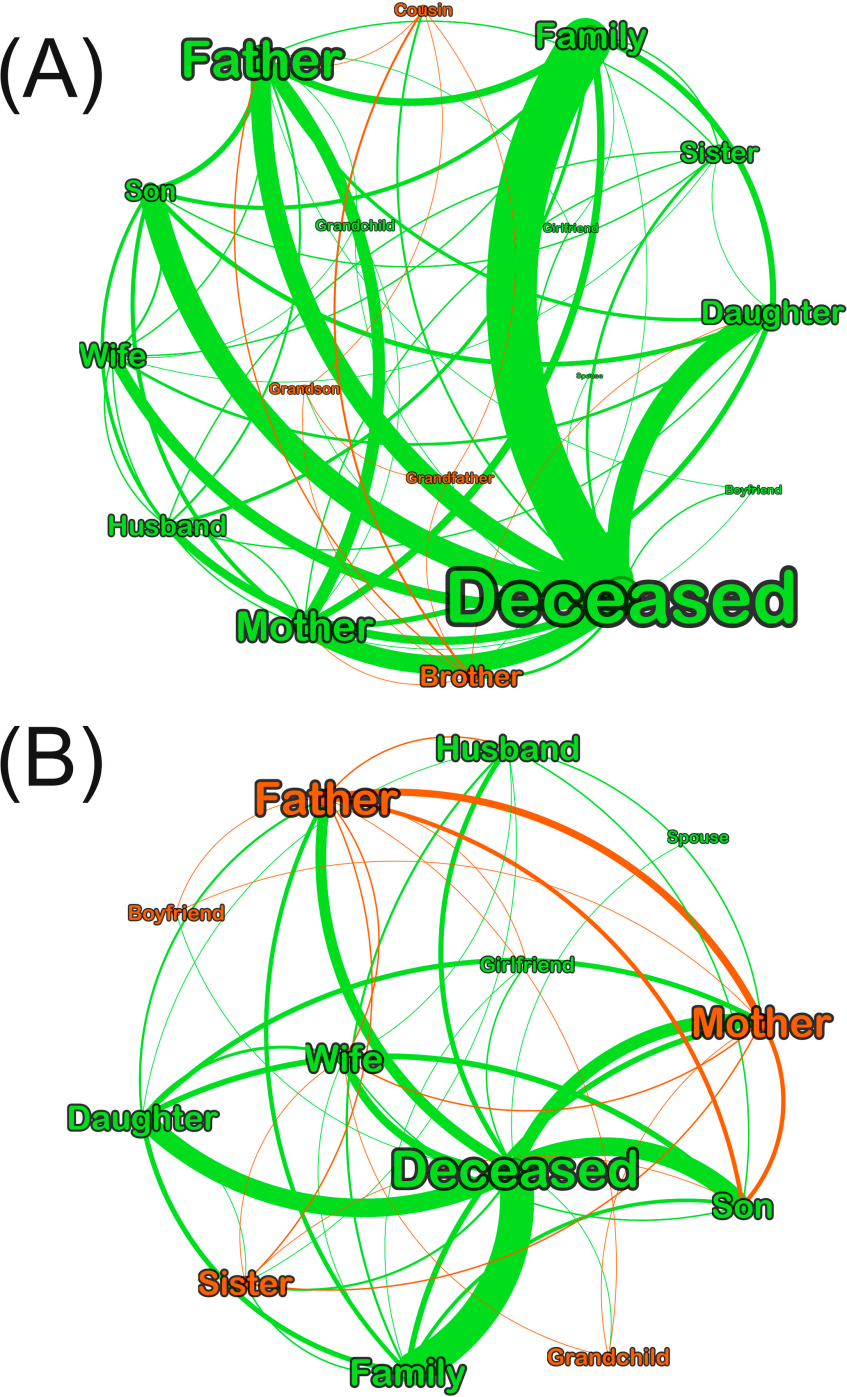
(A) Undirected overall family networks (closeness centrality 1‐0.51; edge thickness 1‐75) and (B) networks of regular people (closeness centrality 1‐0.54; edge thickness 1‐48). Colors indicate different member clusters in each network.

To answer RQ5 and RQ6, Figure 4 illustrates the role of parents as communication hubs in all the videos and in videos of regular people. The larger the label, the higher the closeness centrality, and the thicker the line, the higher the trust (assurance). Although fathers may appear to have the higher closeness centrality, and thus the fastest reach to other family members, the networks of regular people demonstrate that the mother is equally important in this role. Further, the higher trust in videos of regular people is towards sons and daughters.

**Figure 5.**
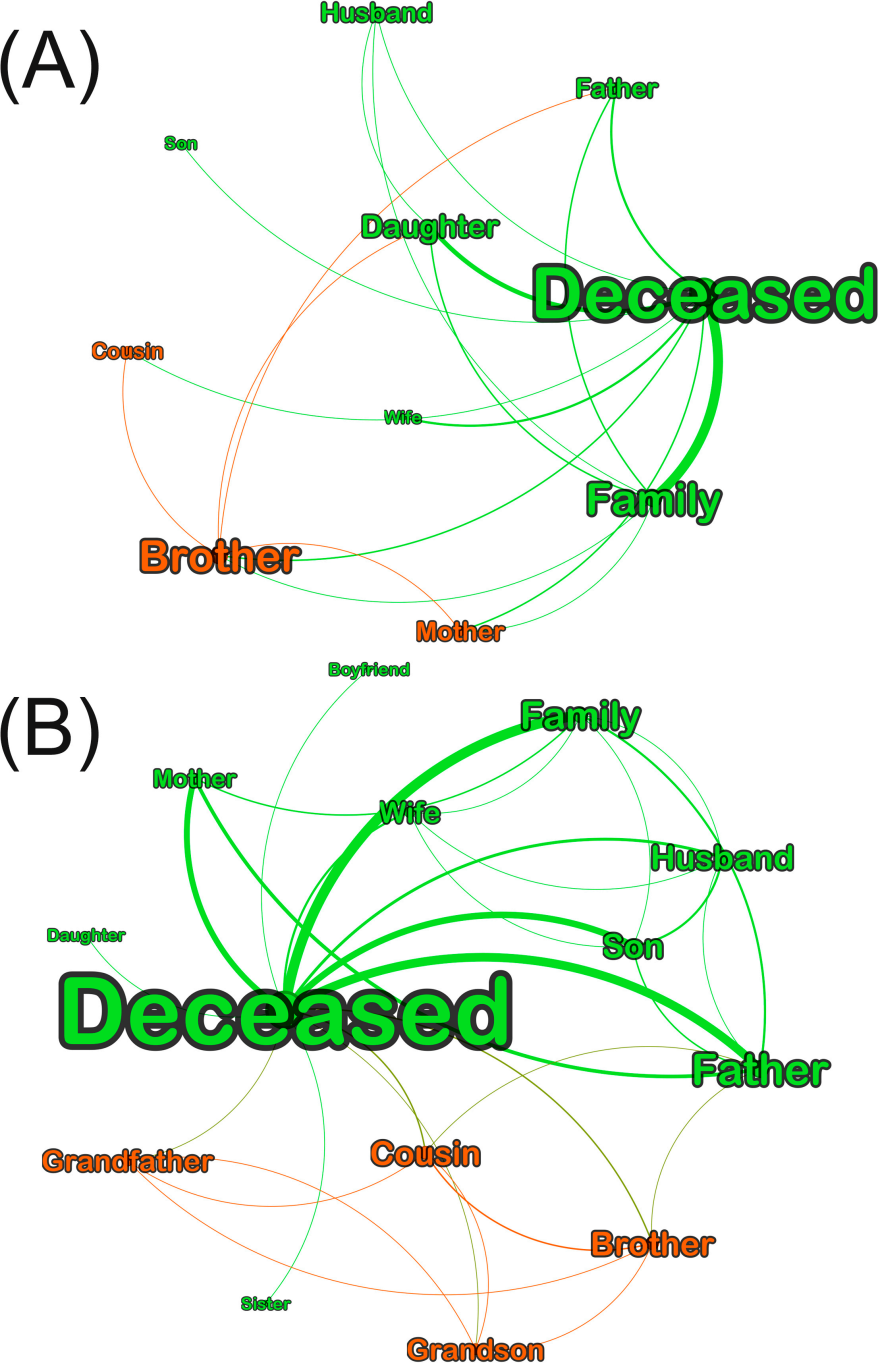
(A) Family networks of celebrities (closeness 1‐0.55; edge thickness 1‐13) and (B) networks of fictional characters (closeness 1‐0.53; edge thickness 1‐14). Colors indicate different member clusters in each network.

On the other hand, Figure 5 highlights that in the separate networks of celebrities and fictional characters, male members of the family such as brothers and husbands are prominent in terms of closeness centrality and trust or assurance. Therefore, horizontal male relations are overrepresented in these networks in contrast to regular people, where vertical relationships including women were most relevant. This complements our results in terms of gender bias representation of the deceased.

## Discussion

### Social Actors in Japanese Peaceful Death

Based on word graphs, the deaths of men, mostly in the adult and older adult age groups, were broadcasted and mourned, even for foreign actors such as Luke Perry, whose last role was in Riverdale, an American streaming series targeting young adults. Based on content and network analysis, the low visibility of women in end-of-life was noted in fictional characters’ death, and to some extent, in that of celebrities. This is not representative of the near gender parity in Japanese deaths [60], or of women patients’ desire for social connection at the end of life [61]. These features were better reflected in videos of regular people’s death, as per Table 3 and Figure 4. In particular, Figure 4B highlights how fathers and mothers have equally fast access to other family members, and how sons and daughters equally show high trust or assurance in the networks.

This bias in gender representation in the other videos may partly occur due to the broader advertising reach for male YouTube viewers [6]. In broader terms, Morioka [62] argues that hegemonic masculinity in Japan, with its goal of ensuring socially dominant roles for men, can twist lay people’s perceptions regarding risks. This may extend to end-of-life scenarios with popular celebrities and fictional characters, wherein the family networks are simplified and the active roles of women as patients, caretakers, and decision makers tend to be hidden from public view.

Beyond the gender binary, a few common people videos in the other or unknown category portrayed queer individuals and their kin, particularly in mourning and honoring the dead. Significant barriers remain in access to health care and family involvement in medical decision-making and care for sexual minorities in Japan [63]. Considering that the recognition of marriage among queer people is in the process of being legalized, the visibility of queer individuals in the videos is still limited but expected to increase in the future. Consequently, it is worthwhile to conduct further studies on how their end-of-life experiences are portrayed in media and how queer health care networks are constructed. Overall, our results provide evidence that studying celebrities and fictional characters has limits in capturing contemporary end-of-life scenarios in Japan. Comparisons of these groups in other topics are also needed.

Content analysis of videos revealed that academics in conferences and open lectures, and a hospice doctor’s YouTube channel provided fora and expert advice on “good death.” They included the process of death, end-of-life care, and alleviating the pain of bereaved families. Although this discourse seems disconnected from other health care scenarios, it is a key connection between experts and the public. Another example of emerging discussions of good death in social media was “*The Crocodile Who Dies After 100 Days*” (Hyaku nichi go ni shinu wani). Although found in a YouTube video, the original was a toon published on Twitter and Instagram between 2019 and 2020. The protagonist is an anthropomorphized crocodile spending time with his friends, wherein the comic panel shows a descending count from 100. The author wished to open discussions about death because “being alive is to die someday” and used Twitter because it was the best way to reach a large audience quickly [64]. These examples show potential in modeling a wider acceptance of public end-of-life conversations among the Japanese public.

### Narratives of Japanese Peaceful Death

Based on keyword graphs, the predominance of cancer in Japanese end-of-life scenarios was reflected in regular people’s videos; however, in general, death due to illness was high. This may partly stem from COVID-19. Although the Japanese government stopped tracking this illness in 2023, the number of general excess deaths and those due to respiratory and circulatory illnesses as well as cancer has increased since the beginning of the pandemic [65]. COVID-19 can be asymptomatic and may also impact multiple organs, triggering respiratory and circulatory issues, and some forms of cancer [66,67]. Considering that young people are also more affected by long COVID-19 than older individuals [68], these trends might be reflected in the videos in our study, where most deaths were not of older adults.

Some researchers estimate that deaths in Japan might be between 1.5 and 3.9 times higher than those reported, and that there is a need to report deaths with more demographic details including age [69]. Our study provides evidence of substantiating this need in the Japanese context, wherein older adults are assumed to account for most of the deaths. We emphasize the need for end-of-life guidelines and care in both sudden death scenarios such as pandemics and long-term disability scenarios for adults of all ages.

Regarding fictional death narratives, Japanese and Western books combined with oral stories and video games appeared frequently. These stories ranged from *kaidan* (old ghost stories) to stories in modern settings, focused on male protagonists. In a previous study, book influencers were important communicators of death on Japanese Twitter (authors, under review). It seems that YouTubers have capitalized on audiovisuals to convey old and new books for monetary and social capital. This may partly explain the bias in targeting male viewers with male-centric death.

### Religion in Japanese Peaceful Death

Buddhism was the most frequent religion in regular people videos, based on content analysis. Buddhist priests, temples, mentions and figures of Buddha, mentions of attaining Buddhahood status in the afterlife, and Buddhist altars for the death (called butsudan) were present in these videos. However, Buddhism was not connected to medical staff or academia. Since most videos did not mention or show a specific Buddhist sect, it is unclear how many people follow new religions masquerading as Buddhism. For example, it is estimated that approximately 3 million people or 2.5% of the Japanese population follows Soka Gakkai [70]. Some estimates of this figure combined with other new religions include 26.47% of the Japanese population [71].

Although less present in the videos, new religions were discussed in terms of the higher self, the astral body, and Wicca (modern European witchcraft). New religions were usually mixed with Buddhism and other minority religions of East Asia, and some videos advertised products. They seem to be diffused across several social media channels, from YouTube, which is popular among people of all ages, to Instagram, which is more popular among young adults. New religions were also inserted in viral topics such as the video about “*The Crocodile Who Dies After 100 Days*.” In Figure 3A, the word “chakra,” is mentioned as a form of individualistic spirituality in videos. Loove [72] mentions that these kinds of new religious practices, rather than depending on specific leadership, are tied to individualized consumer preferences. This consumerism dimension may explain the advertising efforts by practitioners.

In the celebrity videos, the death of former Prime Minister Abe was not connected to religion, according to place classification, annotated religions, and the word graphs. The 10 videos in our data related to Abe were released by Japanese national news media channels such as Ni Tele News and TBS News, and local news media such as SBS News and TV Tokyo BIZ. They focused on the words of Japanese politicians in government offices and common people interviewed in the streets. Conversely, influencers with a considerable fanbase spread the news in neighboring China using pictures and videos, relegating official news media to a less important role [73]. Moreover, a Twitter-based study suggested that the negative emotions elicited by Abe’s death were short-lived [74].

To understand the role of religion in these videos, we must consider that a convergence of interests of some Shinto leaders and state officials to ally when establishing the ideological foundation for Japan. Since the Meiji era, secular politics have been understood as a more civilized form of state formation, so Shinto was redefined by the state as nonreligious immutable customs and traditions of a historically consistent and unified Japan [75]. Here, the apparent nonrepresentation of religion in the celebrity videos uploaded by Japanese news media channels seems to reinforce myths of a hegemonic Japan at the end of life through social learning.

However, we must revisit the example of religion in the Yasukuni Shrine, wherein Abe was among the proponents to enshrine World War II criminals. This act jeopardizes diplomatic relations with neighboring Asian nations that suffered under wartime aggression. Japan is the home of 3.5 million foreign nationals [76], and other ethnic denominations with various spiritual beliefs exist. Therefore, the image of Shinto emphasized by politicians such as Abe might generate discomfort among some Asian patients in Japanese hospitals.

The implication of our results regarding religion for health care providers is that it is not enough to assume that asking simple questions about spirituality at the end-of-life period will capture the beliefs and intentions of an individual and their bereaved family, as previously noted by Uriu et al [30]. Understanding the patient’s concrete spiritual needs beforehand, in collaboration with kin, is recommended. The implication for Japanese media is that they should showcase more religious diversity at the end-of-life period, particularly in the case of celebrities and fictional characters.

### Limitations and Future Studies

This study focused on the term “peaceful death.” Social media discourse is constantly changing. Therefore, it is advisable to monitor other keywords and their combinations to understand other end-of-life scenarios in Japan and other regions.

The data on family networks was limited and did not include friendships. We recommend that more data be gathered from both social media and surveys to better understand family dynamics at the end-of-life period.

### Conclusions

This study examined recent public discussions about peaceful death in Japanese YouTube videos, contributing to recent discourses about health care and end-of-life. From our analyses, we drew several conclusions:

1. Medical experts from academic and hospice contexts address regular people’s deaths in Japan through social media but do not work with religious stakeholders.

2. Most deaths that were discussed were of adult men, with the cause being mostly attributed to illness.

3. Religions are more present in regular peoples’ deaths than in those of celebrities and fictional characters. Buddhism was the most common religion, although various others were also observed.

4. While the deaths of celebrities and fictional characters portray male relatives as more relevant, both men and women were relevant in the deaths of regular people.

5. Networks of regular people indicated the highest assurance, fastest access to other family members, and the highest density and complexity. Fathers and mothers had the fastest access to other family members during peaceful death. Sons and daughters showed the highest assurance during peaceful death.

## Supplementary material

10.2196/81861 Multimedia Appendix 1 Classification of individuality.

10.2196/81861 Multimedia Appendix 2 Narratives of death.

10.2196/81861 Multimedia Appendix 3 Intercoder agreement.

10.2196/81861 Multimedia Appendix 4 Network measurements.

10.2196/81861 Multimedia Appendix 5 Findings from the death narratives.

## Abbreviations

RQ: research question
TBS: Tokyo Broadcasting System

## References

[1] (2022). Number of deaths. Vital statistics [article in japanese]. Portal Site of Official Statistics of Japan.

[2] Nakazawa K, Kizawa Y, Maeno T (2014). Palliative care physicians’ practices and attitudes regarding advance care planning in palliative care units in Japan: a nationwide survey. Am J Hosp Palliat Care.

[3] Long SO (2005). Final Days: Japanese Culture and Choice at the End of Life.

[4] Hattori K, McCubbin MA, Ishida DN (2006). Concept analysis of good death in the Japanese community. J Nurs Scholarsh.

[5] Murphy AK, Jerolmack C, Smith D (2021). Ethnography, data transparency, and the information age. Annu Rev Sociol.

[6] Kemp S Digital 2024: Japan.

[7] (2023). Share of people who use youtube in Japan in fiscal year 2024, by age group. Statista.

[8] Maruyama K, Hayashi F, Kamisasa H (1981). A multivariate analysis of Japanese attitude toward life and death. Behaviormetrika.

[9] Nagamine T (1988). Attitudes toward death in rural areas of Japan. Death Stud.

[10] Tashiro J (1993). A study on regarding death—a comparison between medical professionals and general adults [Article in Japanese]. Bioethics.

[11] Long SO (2003). Becoming a cucumber: Culture, nature, and the good death in Japan and the United States. J Jpn Stud.

[12] Miyashita M, Kawakami S, Kato D (2015). The importance of good death components among cancer patients, the general population, oncologists, and oncology nurses in Japan: patients prefer “fighting against cancer”. Support Care Cancer.

[13] Morooka R (2017). Death and “nuisance”—the current state of views on life and death in modern Japan [Article in Japanese]. Relig Soc.

[14] Gould H (2023). From a ‘good death’ to a ‘calm heart’: Buddhist retailing meets self-care in contemporary Japan. J Contemp Relig.

[15] Sueki H (2015). The association of suicide-related Twitter use with suicidal behaviour: a cross-sectional study of young internet users in Japan. J Affect Disord.

[16] Ueda M, Mori K, Matsubayashi T, Sawada Y (2017). Tweeting celebrity suicides: users’ reaction to prominent suicide deaths on Twitter and subsequent increases in actual suicides. Soc Sci Med.

[17] Hidemichi K (2021). コロナウイルスによる 「死」 をメディアはどう伝えるのか―差別や偏見をなくす視点からの考察 [Article in Japanese]. Risk Manag Stud.

[18] Anderson WC (2024). Curating colonization: on sharing visuals of the dead. Logic(s) Magazine.

[19] Foltyn JL (2008). Dead famous and dead sexy: popular culture, forensics, and the rise of the corpse. Mortality (Abingdon).

[20] Kennedy J (2010). DON’T You Forget About ME: An exploration of the “Maddie Phenomenon” on YouTube. Journal Stud.

[21] Radford SK, Bloch PH (2013). Consumers’ online responses to the death of a celebrity. Mark Lett.

[22] Bragazzi NL, Watad A, Brigo F, Adawi M, Amital H, Shoenfeld Y (2017). Public health awareness of autoimmune diseases after the death of a celebrity. Clin Rheumatol.

[23] VanderKnyff J, Friedman DB, Tanner A (2015). Framing life and death on YouTube: the strategic communication of organ donation messages by organ procurement organizations. J Health Commun.

[24] Pentaris P, Yerosimou M (2020). The functional role of music in communicating death through/in YouTube videos. J Edu. Cult Soc.

[25] Pentaris P, Yerosimou M (2015). The role of background music in the experience of watching YouTube videos about death and dying. J Educ Cult Soc.

[26] Giménez-Llort L (2021). An ethnography study of a viral YouTube educational video in Ecuador: dealing with death and grief in times of COVID-19. Front Psychiatry.

[27] Eriksson Krutrök M (2021). Algorithmic closeness in mourning: vernaculars of the hashtag #grief on TikTok. Soc Media Soc.

[28] Karabulut MH (2022). Virtual Grieving in the Times of COVID-19 [Master’s thesis]. https://open.metu.edu.tr/handle/11511/101216.

[29] Forrest RA (2019). An analysis of English-language YouTube videos related to Minamata disease: prior to the release of the Hollywood film “Minamata" [Article in Japanese]. Hiroshima keizai daigaku kenkyuu ronshuu / Research papers of the Hiroshima University of Economics.

[30] Uriu D, Toshima K, Manabe M (2021). Generating the presence of remote mourners: a case study of funeral webcasting in japan. https://dl.acm.org/doi/abs/10.1145/3411764.3445617.

[31] Mou Y, Lan J, Huang Y (2025). Good night versus goodbye? Comparing the mourning remarks of virtual and human uploaders through a data-mining approach. New Media Soc.

[32] Trusson R (2024). ‘Shinu shika nai’–‘There is Nothing to do but to Die’: Contextualising the Rising Young Female Suicide Rate in Japan [Masters thesis]. https://etheses.dur.ac.uk/15311/.

[33] Widmer ED, Aeby G, Sapin M (2013). Collecting family network data. Int Rev Sociol.

[34] Burns CM, Abernethy AP, Dal Grande E, Currow DC (2013). Uncovering an invisible network of direct caregivers at the end of life: a population study. Palliat Med.

[35] Leonard R, Horsfall D, Noonan K (2015). Identifying changes in the support networks of end-of-life carers using social network analysis. BMJ Support Palliat Care.

[36] Leonard R, Horsfall D, Rosenberg J, Noonan K (2020). Carer experience of end-of-life service provision: a social network analysis. BMJ Support Palliat Care.

[37] Cornwell B, Laumann EO, Alwin DF, Felmlee DH, Kreager DA (2018). Social Networks and the Life Course: Integrating the Development of Human Lives and Social Relational Networks.

[38] Nojiri Y (1974). Family social network in modern Japan: an application of path analysis [Article in Japanese]. Jpn Sociol Rev.

[39] Maeda N, Meguro Y (1990). A comparative analysis of social network patterns of urban families across social classes [ Article in Japanese]. J Fam Sociol.

[40] Iwasaki M, Otani T, Ohta A, Yosiaki S, Kuroiwa M, Suzuki S (2002). Rural-urban differences in sociodemographic, social network and lifestyle factors related to mortality of middle-aged Japanese men from the Komo-Ise cohort study. J Epidemiol.

[41] Ishiguro I (2018). Changes in core network size in Japan: comparisons between the 1990s and 2010s. Soc Networks.

[42] Miyashita J, Yamamoto Y, Shimizu S (2019). Association between social networks and discussions regarding advance care planning among Japanese older adults. PLOS ONE.

[43] (2023). YouTube data tools. Digital Methods.

[44] Park HW, Lim YS (2020). Do North Korean social media show signs of change?: An examination of a YouTube channel using qualitative tagging and social network analysis. J Contemp East Asia.

[45] Vargas Meza X, Oikawa M (2024). Japanese perception of brain death and implications for new medical technologies: quantitative and qualitative social media analysis. JMIR Form Res.

[46] Yasuoka MK (2015). Organ Donation in Japan: A Medical Anthropological Study.

[47] Vargas Meza X, Oikawa M (2024). Japanese perception of organ donation and implications for new medical technologies: quantitative and qualitative social media analyses. JMIR Form Res.

[48] Higuchi K (2016). A two-step approach to quantitative content analysis: KH Coder tutorial using Anne of Green Gables (Part I). Ritsumeikan Soc Sci Rev.

[49] Vijaymeena MK, Kavitha K (2016). A survey on similarity measures in text mining. MLAIJ.

[50] Higuchi K (2016). KH coder 3 reference manual. https://khcoder.net/en/manual_en_v3.pdf.

[51] Cadima R, Ojeda Rodríguez J, Monguet Fierro JM (2012). Social networks and performance in distributed learning communities. Edu Tech Society.

[52] Song H, Eveland WP (2015). The structure of communication networks matters: how network diversity, centrality, and context influence political ambivalence, participation, and knowledge. Polit Commun.

[53] Das K, Samanta S, Pal M (2018). Study on centrality measures in social networks: a survey. Soc Netw Anal Min.

[54] Xanat VM, Shigen S, Hayashi Y, Takada H, Marutschke DM, Alvarez C (2023). Semantic network analysis of a learning task among Japanese students of psychology.

[55] Yamagishi T (2011). Trust: The Evolutionary Game of Mind and Society.

[56] Bastian M, Heymann S, Jacomy M Gephi: an open source software for exploring and manipulating networks.

[57] Gephi.

[58] (2019). Internet research: ethical guidelines 3.0. https://aoir.org/reports/ethics3.pdf.

[59] (2021). Ethical guidelines for medical and biological research involving human subjects. https://www.mext.go.jp/content/20250325-mxt_life-000035486-01.pdf.

[60] (2024). Vital statistics report: occupational and industrial aspects general mortality. Portal Site of Official Statistics of Japan.

[61] Hirakawa Y, He Y, Chiang C, Aoyama A (2019). Gender differences in wishes and feelings regarding end-of-life care among Japanese elderly people living at home. J Rural Med.

[62] Morioka R (2014). Gender difference in the health risk perception of radiation from Fukushima in Japan: the role of hegemonic masculinity. Soc Sci Med.

[63] Otani H, Morita T, Kim H (2024). Medical care needs and experiences of LGBTQ populations in Japan. medRxiv.

[64] (2020). The author of “The Crocodile Who Will Die in 100 Days” is getting a lot of attention on social media ‘the ending is already decided’ [Article in Japanese]. Oricon News.

[65] (2024). Excess and exiguous deaths dashboard in Japan. Ministry of Health, Labour and Welfare of Japan.

[66] Moslehi N, Jahromy MH, Ashrafi P (2022). Multi-organ system involvement in coronavirus disease 2019 (COVID-19): a mega review. J Family Med Prim Care.

[67] Mitrofanova L, Makarov I, Goncharova E (2023). High risk of heart tumors after COVID-19. Life (Basel).

[68] Choudhury NA, Mukherjee S, Singer T (2025). Neurologic manifestations of long COVID disproportionately affect young and middle-age adults. Ann Neurol.

[69] Adam D (2022). The pandemic’s true death toll: millions more than official counts. Nature.

[70] McLaughlin L (2018). Soka Gakkai’s Human Revolution: The Rise of a Mimetic Nation in Modern Japan.

[71] (2024). National / regional profiles. Association of Religion Data Archives.

[72] Loov M (2024). The New Age Movement.

[73] Qi H (2022). Analysis of the transmission of international breaking news on social media platforms from the perspective of tipping point theory—taking the assassination of former Japanese Prime Minister Shinzo Abe as an example. J Educ Hum Soc Sci.

[74] Takagi MN (2023). The effects of political martyrdom on election results: the assassination of Abe. J Data Min Digit Humanit.

[75] Anderson E (2014). Christianity and Imperialism in Modern Japan.

[76] (2024). Number of foreign residents in japan hits record 3.5 million as of june [article in japanese]. Immigration Services Agency, Ministry of Justice (Japan).

[77] Vargas Meza X, Oikawa M Anonymised youtube dataset related to peaceful death in japan. https://zenodo.org/records/18168743.

