# Supplementary material for: Peaceful Death in Japanese YouTube Videos: Content and Network Analysis
Source: JMIR Form Res. 2026 Mar 13;10:e81861. doi: 10.2196/81861 (PMC12986788; doi:10.2196/81861)
Supplement: Multimedia Appendix 1 [file formative-v10-e81861-s001.docx]

**Appendix 1.** Classification of individuality

| Actor Type | Subtype | Description |
| --- | --- | --- |
| Medical Staff | Doctor | Surgeons, doctors, therapists |
|  | Medical student | Medicine and nursing students |
|  | Nurse | Nursing professionals |
|  | Other medical staff | Secretaries, donor coordinators, hospital personnel |
| Patients and Relatives | Donor | Organ/tissue donors |
|  | Recipient | Organ/tissue recipients |
|  | Donor relative | Organ/tissue donor relatives |
|  | Recipient relative | Organ/tissue recipient relatives |
|  | Other deceased |  |
|  | Other deceased relative |  |
| Others | Association | Organizations for dignified death, disease prevention, organ donation, etc. |
|  | Citizen | Common people |
|  | Government | Government officials, judges, police |
|  | Media | Journalists and media companies |
|  | Religion | Religious heads, preachers, devotees |
|  | Education | Non-medical researchers, educators, students |
|  | Unknown | Actors that could not be classified in other categories. |
