# Supplementary material for: Peaceful Death in Japanese YouTube Videos: Content and Network Analysis
Source: JMIR Form Res. 2026 Mar 13;10:e81861. doi: 10.2196/81861 (PMC12986788; doi:10.2196/81861)
Supplement: Multimedia Appendix 2 [file formative-v10-e81861-s002.docx]

**Appendix 2.** Types of death and end of life narratives in Japan based on ^a^Nagamine (1998), ^b^Long (2003), ^c^Long (2005), ^d^Yasuoka (2015) and ^e^authors.

| **Japanese** | **English** | **Explanation** |
| --- | --- | --- |
| 命 | Life^a^ | Death is life, there are no clear divisions between life and death |
| 戦死 | War death^b^ | Death occurred during a war conflict |
| 殺人 | Homicide^b^ |  |
| 自殺 | Suicide^b^ |  |
| 事故死 | Accidental death^b^ |  |
| 説明・理由あり | Explanation^c^ | Presence of an explanation for death |
| 老衰 | Senile death^c^ | Gradual death, usually in elderly age |
| ぽっくり死 | Sudden death^c^ | Painless and sudden death |
| 安らかな死 | Peaceful death^c^ | Death that is peaceful, painless, being cared for |
| 延命治療死 | Treatment death^c^ | Death while receiving medical treatment. |
| 畳の上の死 | Death over a rice mat^c^ | Death in one’s home |
| 信頼死 | Reliant death^c^ | The patient trusts their caretakers |
| 迷惑な死 | Burdensome death^c^ | A death that causes burdens to kin |
| 死に目に会う | Look the dead in the eye^c^ | Death with company |
| 孤独な死 | Lonely death^c^ |  |
| 自然な死 | Natural death^c^ | Death of natural causes |
| 尊厳死 | Death with dignity^c^ |  |
| 安楽死 | Euthanasia^c^ |  |
| 死を受け入れる | Accepting death^c^ |  |
| 頑張る | Doing your best^d^ | The patient is doing/did their best during their struggle with illness. |
| 病死 | Illness death^e^ | Death due to a specific illness |
| ありがたい | Gratitude^e^ | Having gratitude during end-of-life |
| 痛み | Pain^e^ | Death is painful |
| 穏やかな | Calm death^e^ |  |
