# Supplementary material for: Peaceful Death in Japanese YouTube Videos: Content and Network Analysis
Source: JMIR Form Res. 2026 Mar 13;10:e81861. doi: 10.2196/81861 (PMC12986788; doi:10.2196/81861)
Supplement: Multimedia Appendix 3 [file formative-v10-e81861-s003.docx]

**Appendix 3.** Intercoder agreement per family member

| **Member** | Boyfriend | Brother | Cousin | Daughter | Father | Grandchild | Grandfather | Grandson | Girlfriend | Husband | Mother | Sister | Son | Spouse | Wife |
| --- | --- | --- | --- | --- | --- | --- | --- | --- | --- | --- | --- | --- | --- | --- | --- |
| **Agreement** | 0.98 | 0.98 | 1 | 0.90 | 0.86 | 1 | 1 | 1 | 1 | 0.93 | 0.83 | 0.97 | 0.90 | 1 | 0.97 |
