# Supplementary material for: Peaceful Death in Japanese YouTube Videos: Content and Network Analysis
Source: JMIR Form Res. 2026 Mar 13;10:e81861. doi: 10.2196/81861 (PMC12986788; doi:10.2196/81861)
Supplement: Multimedia Appendix 4 [file formative-v10-e81861-s004.docx]

**Appendix 4.** Network measurements and their implications for this study.

| **Measure** | **Definition** | **Implication** |
| --- | --- | --- |
| Nodes | Family members in the network | Few nodes indicate a small family |
| Edges | Family members who appeared in the same video | No edges imply disconnection, many edges imply assurance |
| Average Weighted Degree | Average number of edges a family member has with others | Indicates that a family member has greater access to other members and may have a high participation when taking decisions |
| Average harmonic closeness | Average number of steps a family member needs to reach all the other members | Indicates that a family member has fast access to other members and low participation in family decisions |
| Average betweenness | Number of shortest paths that tie family members by passing through a specific family member | Indicates a family member who is a bridge between member groups |
| Average clustering coefficient | Average of number of edges between members of a group | Implies assurance at the member level |
| Average path lenght | Average of the distance between members | Proximity of members |
| Density | Total edges divided by the number of all possible edges | Indicates overall participation and assurance |
| Diameter | Average of the maximum distance between members | Indicates overall socialization |
| Modularity | Strength of the division between member groups | Identification of distinctive member groups in a family |
| Triangles | Number of groups of three members tied to each other | Implies transitivity of information and complexity of the family structure |
| Connected components | Group of members that has at least one connection to other groups | Indicates family cohesiveness |
