# Supplementary material for: Peaceful Death in Japanese YouTube Videos: Content and Network Analysis
Source: JMIR Form Res. 2026 Mar 13;10:e81861. doi: 10.2196/81861 (PMC12986788; doi:10.2196/81861)
Supplement: Multimedia Appendix 5 [file formative-v10-e81861-s005.docx]

Appendix 5. Overlapping death narratives in YouTube videos.

| **Narrative** | **Regular videos**  **(n=220)**  n/mean | **Celebrities videos**  **(n=122)**  n/mean | **Fictional videos**  **(n=115)**  n/mean | **Total videos**  **(n=457)**  n/mean |
| --- | --- | --- | --- | --- |
| Life | 22 / 0.10 | 2 / 0.01 | 5 / 0.04 | 29 / 0.06 |
| War death | 10 / 0.04 | 0 / 0.00 | 13 / 0.11 | 23 / 0.05 |
| Homicide | 21 / 0.10 | 13 / 0.10 | 24 / 0.20 | 58 / 0.12 |
| Suicide | 16 / 0.07 | 12 / 0.10 | 8 / 0.07 | 36 / 0.07 |
| Accidental death | 19 / 0.08 | 7 / 0.05 | 9 / 0.07 | 35 / 0.07 |
| Explanation | 100 / 0.45 | 61 / 0.50 | 61 / 0.53 | 222 / 0.48 |
| Senile death | 13 / 0.06 | 3 / 0.02 | 2 / 0.01 | 18 / 0.04 |
| Sudden death | 3 / 0.01 | 0 / 0.00 | 0 / 0.00 | 3 / 0.00 |
| Peaceful death | 32 / 0.14 | 21 / 0.17 | 14 / 0.12 | 67 / 0.14 |
| Treatment death | 39 / 0.17 | 12 / 0.10 | 8 / 0.07 | 59 / 0.13 |
| Death over a rice mat | 18 / 0.08 | 3 / 0.02 | 6 / 0.05 | 27 / 0.06 |
| Reliant death | 2 / 0.01 | 0 / 0.00 | 1 / 0.00 | 3 / 0.00 |
| Burdensome death | 2 / 0.01 | 0 / 0.00 | 0 / 0.00 | 2 / 0.00 |
| Look the dead in the eye | 24 / 0.11 | 2 / 0.01 | 6 / 0.05 | 32 / 0.07 |
| Lonely death | 13 / 0.06 | 1 / 0.00 | 1 / 0.00 | 15 / 0.03 |
| Natural death | 8 / 0.03 | 0 / 0.00 | 1 / 0.00 | 9 / 0.02 |
| Death with dignity | 4 / 0.01 | 0 / 0.00 | 0 / 0.00 | 4 / 0.00 |
| Euthanasia | 21 / 0.10 | 0 / 0.00 | 10 / 0.08 | 31 / 0.06 |
| Accepting death | 6 / 0.02 | 2 / 0.01 | 4 / 0.03 | 12 / 0.02 |
| Doing your best | 9 / 0.04 | 2 / 0.01 | 0 / 0.00 | 11 / 0.02 |
| Illness death | 53 / 0.24 | 30 / 0.24 | 18 / 0.15 | 101 / 0.22 |
| Gratitude | 14 / 0.06 | 2 / 0.01 | 2 / 0.01 | 18 / 0.04 |
| Pain | 8 / 0.03 | 0 / 0.00 | 2 / 0.01 | 10 / 0.02 |
| Calm death | 13 / 0.06 | 1 / 0.00 | 0 / 0.00 | 14 / 0.03 |
| Total narratives | 470 / 2.13 | 174 / 1.42 | 195 / 1.70 | 839 / 1.83 |
